# Supplementary material for: CS12192, a Novel JAK3/JAK1/TBK1 Inhibitor, Synergistically Enhances the Anti-Inflammation Effect of Methotrexate in a Rat Model of Rheumatoid Arthritis
Source: Int J Mol Sci. 2022 Nov 2;23(21):13394. doi: 10.3390/ijms232113394 (PMC9658750; doi:10.3390/ijms232113394)
Supplement: Supplementary file 1 [file ijms-23-13394-s001.zip › ijms-1946696-supplementary.pdf]

CS12192, a novel JAK3/JAK1/TBK1 inhibitor, synergistically enhances the anti-inflammation effect of methotrexate on a rat model of rheumatoid arthritis

Zhengyu Fang [1,2,#], Yiping Hu [1,#], Jiajing Dai [3], Lianhua He [1], Juan He [1], Bihua Xu [1], Xinle Han [3], Fubo Zhong [2,3], Hui-Yao Lan [4,*] and Qingwen Wang [1,*]

[1] Department of Rheumatism and Immunology, Peking University Shenzhen Hospital. Shenzhen key Laboratory of Inflammatory and Immunology Diseases, Shenzhen, Guangdong Province, China

[2] Department of Pathology, Peking University Shenzhen Hospital, Guangdong Province, China

[3] Clinical Research Institute, Shenzhen Peking University - The Hong Kong University of Science and Technology Medical Center, Guangdong Province, China.

[4] Department of Medicine and Therapeutics, Li Ka Shing Institute of Health Sciences, and Lui Che Woo Institute of Innovative Medicine, The Chinese University of Hong Kong, Hong Kong, China.

* Correspondence: Qingwen Wang, wangqingwen@pkuszh.com; Hui-Yao Lan, hylan@cuhk.edu.hk

[#] These authors contributed equally to this work

Supplementary Figure Lengends

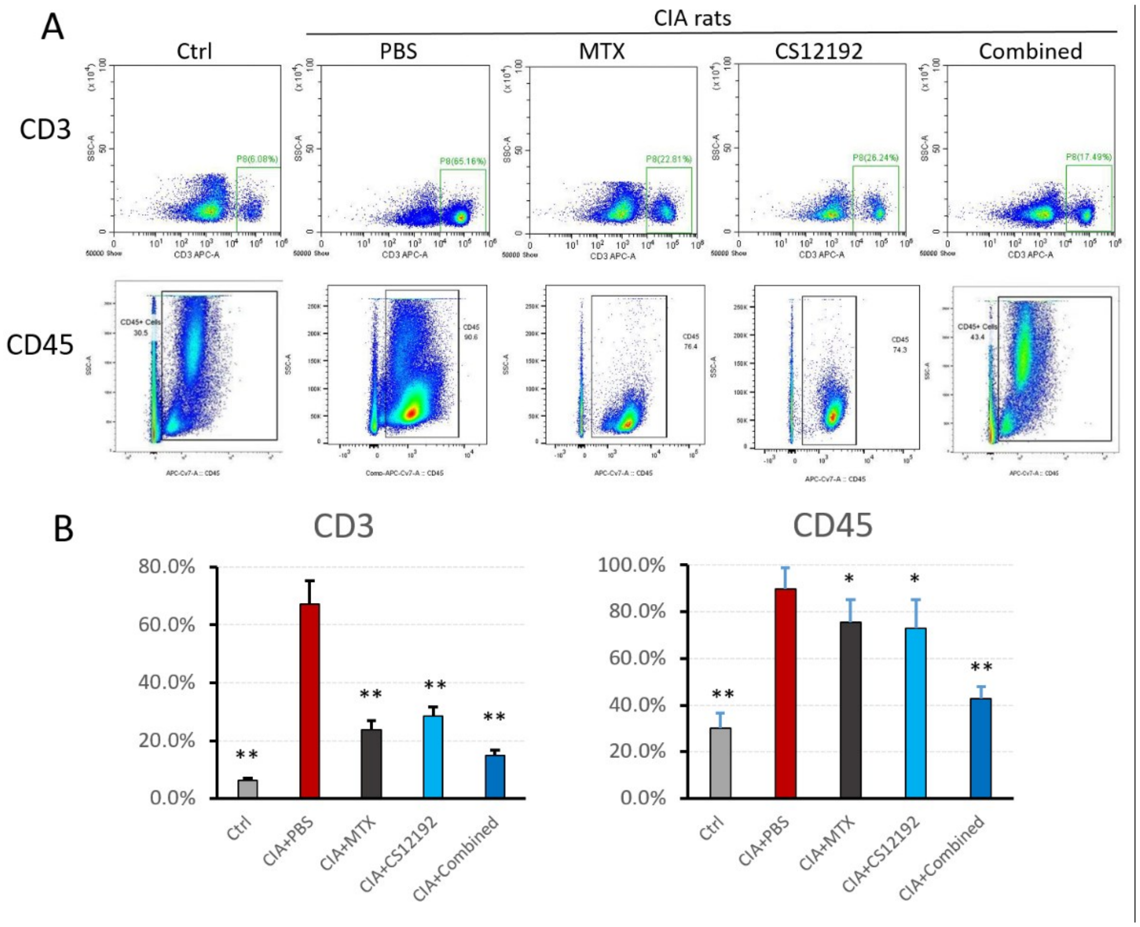

Figure S1. Effects of MTX, CS12192, or combined treatments on immune cells: (A) Representative flow cytometric plots showing CD45+ or CD3+ T cells in single live cell suspension from peripheral blood of rats in different groups. Flow cytometry results showing the percentage of CD45+ or CD3+ T cells. (B) Data are presented as means ± SEM. Statistical significance was calculated by Student's t-test: **P < 0.01, *P < 0.05.

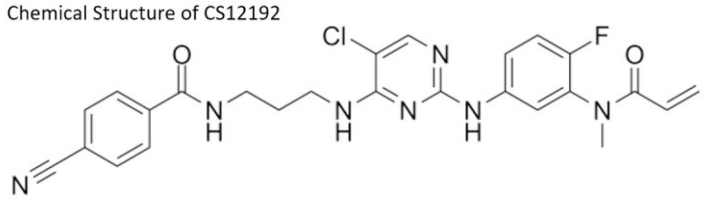

Figure S2. Molecular structure of CS12192.
